# Medical Association of Macon

**Published:** 1871-05

**Authors:** 


### MEDICAL ASSOCIATION OF MACON.

We lay before our readers, in this number, the proceedings, with preamble and resolutions, of the Medical Association of Macon, transmitted to us by its Secretary, Dr. Wm. R. Burgess, for publication. We express our gratitude, as individual members of the Georgia Medical Association, for this prompt and high professional action by the profession of Macon, in sustaining the previous action of the State Association and the protest of those members present at its late meeting, who placed themselves in the breach against the unconstitutional and revolntionary action of Dr. Logan and his party. We feel called upon, however, in justice to our Macon friends, as well as to the Georgia Medical Association, to dissent—with the greatest kindness—to that portion of one of their resolutions in reference to Dr. H. V. M. Miller. While Dr. Miller may not, so far as we know, have signed the memorial with his own hand, his name appeared in connection with the other members of the Faculty, and was there suffered to remain until the present moment, notwithstanding one of the aggrieved parties addressed him a kind letter asking if he did sign it, to which he has never replied; and wherever the memorial has been read, the charges therein contained have had the influence of his name and weight of character. If he did not sign it, he has either loaned the use of his name, or some one or more are guilty of forgery. We are satisfied our Macon friends are not aware of the reason why Dr. Miller did not disclaim having any connection with the memorial until after the passage of the "Logan preamble and resolution." Dr. Miller, however, no doubt well remembers, and we have not forgotten, *the reason.*

